# Clinical and genetic characterization of patients with Pierre Robin sequence and spinal disease: review of the literature and novel terminal 10q deletion

**DOI:** 10.1007/s00381-020-04642-2

**Published:** 2020-05-12

**Authors:** Anudeep Yekula, Connor Grant, Mihir Gupta, David R. Santiago-Dieppa, Pate J. Duddleston, David Gonda, Michael Levy

**Affiliations:** 1 Department of Neurosurgery, Massachusetts General Hospital, Boston, MA USA; 2 Department of Neurosurgery, University of California San Diego, La Jolla, CA USA; 3 San Diego, CA USA; 4 School of Medicine, Mercer University, Savannah, GA USA; 5 Department of Pediatric Neurosurgery, Rady Children’s Hospital, San Diego, CA USA

**Keywords:** Pierre Robin sequence, Spine, Tethered cord, Deformity, Molecular genetics, Genomics, Molecular basis of disease

## Abstract

**Introduction:**

The Pierre-Robin sequence (PRS) is a pattern of congenital facial abnormalities comprising micrognathia, glossoptosis, and airway obstruction. Associated spinal pathologies have rarely been reported with PRS.

**Methods:**

We explore the molecular genetic basis of this association through a systematic review of spinal disease in patients with PRS. We also present an illustrative case of a PRS patient with tethered cord in the setting of chromosome 10q terminal deletion.

**Results:**

Our systematic literature review of spinal disease in patients with PRS revealed several patterns in the underlying genetic syndromes causing these conditions to co-occur. These principles are illustrated in the case of a 6-month-old female with PRS and a 14.34-Mb terminal deletion of chromosome 10q, who was found to have a sacral dimple during a routine outpatient checkup. Magnetic resonance imaging of the spine revealed a lumbar syrinx associated with tethered spinal cord. Surgical de-tethering was undertaken, with subsequent improvement in motor function and decrease in the size of the syrinx. The deletion of chromosome 10q in our patient had not previously been described in association with tethered cord or PRS.

**Conclusion:**

Spinal pathologies are understudied contributors to disease burden in patients with PRS. The range of predisposing syndromes and mutations in patients with both PRS and spinal disorders remains poorly characterized but may be more defined than previously conceived. Clinical screening is most critical during neonatal and adolescent developmental periods with continued neurological assessment. This study emphasizes the need for early genetic testing and counseling in this patient population, in parallel with research efforts to develop molecular classifications to guide clinical management.

**Electronic supplementary material:**

The online version of this article (10.1007/s00381-020-04642-2) contains supplementary material, which is available to authorized users.

## Background

The Pierre Robin sequence (PRS), also known as Robin sequence, is a pattern of congenital facial abnormalities comprising micrognathia, glossoptosis, and airway obstruction [1]. The reported incidence varies widely with an approximate occurrence of 1 in 8500 to 14,000 births. Approximately 50% of PRS cases are isolated (non-syndromic), while the remainder are associated with additional anomalies such as a genetic or acquired syndrome [2–5].

PRS is most commonly associated with hearing loss, dysmorphic facial features, global developmental delay/intellectual disability, and/or congenital heart defects [4]. Spinal pathologies have rarely been reported in association with PRS, often co-occurring with other congenital anomalies [6–22]. The molecular genetic and clinical characteristics of spinal disease in PRS remain poorly characterized. We thus performed a systematic review of spinal disease in patients with PRS. We additionally report a case of a PRS patient presenting with tethered cord and lumbar syrinx in the setting of chromosome 10q terminal deletion.

## Illustrative case

A 6-month-old female with a history of PRS was found to have a sacral dimple during a routine outpatient checkup. Neurological examination was notable for diffuse hypotonia and global developmental delay, but without focal deficit, incontinence, or prior urinary tract infections. No other cutaneous stigmata were noted. Medical history included premature birth at 31 weeks with grade I intraventricular hemorrhage. She had multiple congenital abnormalities including micrognathia, glossoptosis, and airway obstruction characteristic of PRS, as well as cleft palate, strabismus, patent ductus arteriosus, mild dilation of the aortic sinuses, clinodactyly of the fifth finger, and syndactyly involving the bilateral second and third toes.

Prior microarray analysis as a neonate had shown a 14.34 Mb terminal deletion of chromosome 10q. The deletion extended from band 10q26.11 to 10q26.3, comprising 112 genes including 62 OMIM genes (Supplementary Table 1). Genetic counseling was pursued, and the patient’s parents declined additional familial karyotyping.

**Fig. 1:**
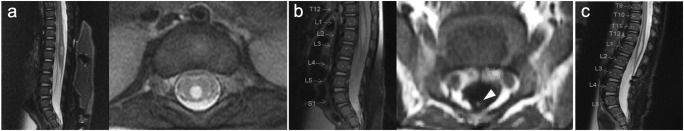
Magnetic resonance imaging of the lumbosacral region. **a** Sagittal and axial T2-weighted scans at 6 months of age showing a syrinx of the conus medullaris. **b** Sagittal T2-weighted and axial T1-weighted scans at 12 months of age showing enlargement of the syrinx, borderline low-lying conus medullaris, and fatty infiltration of the filum terminale (arrowhead) consistent with tethered cord. **c** Sagittal T2-weighted scan 8 months after release of tethered cord showing decreased syrinx size

Magnetic resonance imaging (MRI) of the spine with and without contrast revealed a syrinx of the conus medullaris without other abnormalities (Fig. 1a). This was managed expectantly with repeat MRI at 12 months of age, which showed expansion of the syrinx and fatty infiltration of the filum terminale consistent with tethered spinal cord (Fig. 1b). Surgical release of the tethered cord was thus undertaken. The procedure was uneventful and the patient recovered well postoperatively. Follow-up MRI 8 months postoperatively showed slight decrease in the size of the syrinx (Fig. 1c). One year postoperatively, neurological examination remained nonfocal without any deficits referable to the tethered cord or surgical procedure. Motor and cognitive function were improved compared to initial presentation but with ongoing delay in meeting respective milestones.

## Systematic literature review

### Methods

**Fig. 2:**
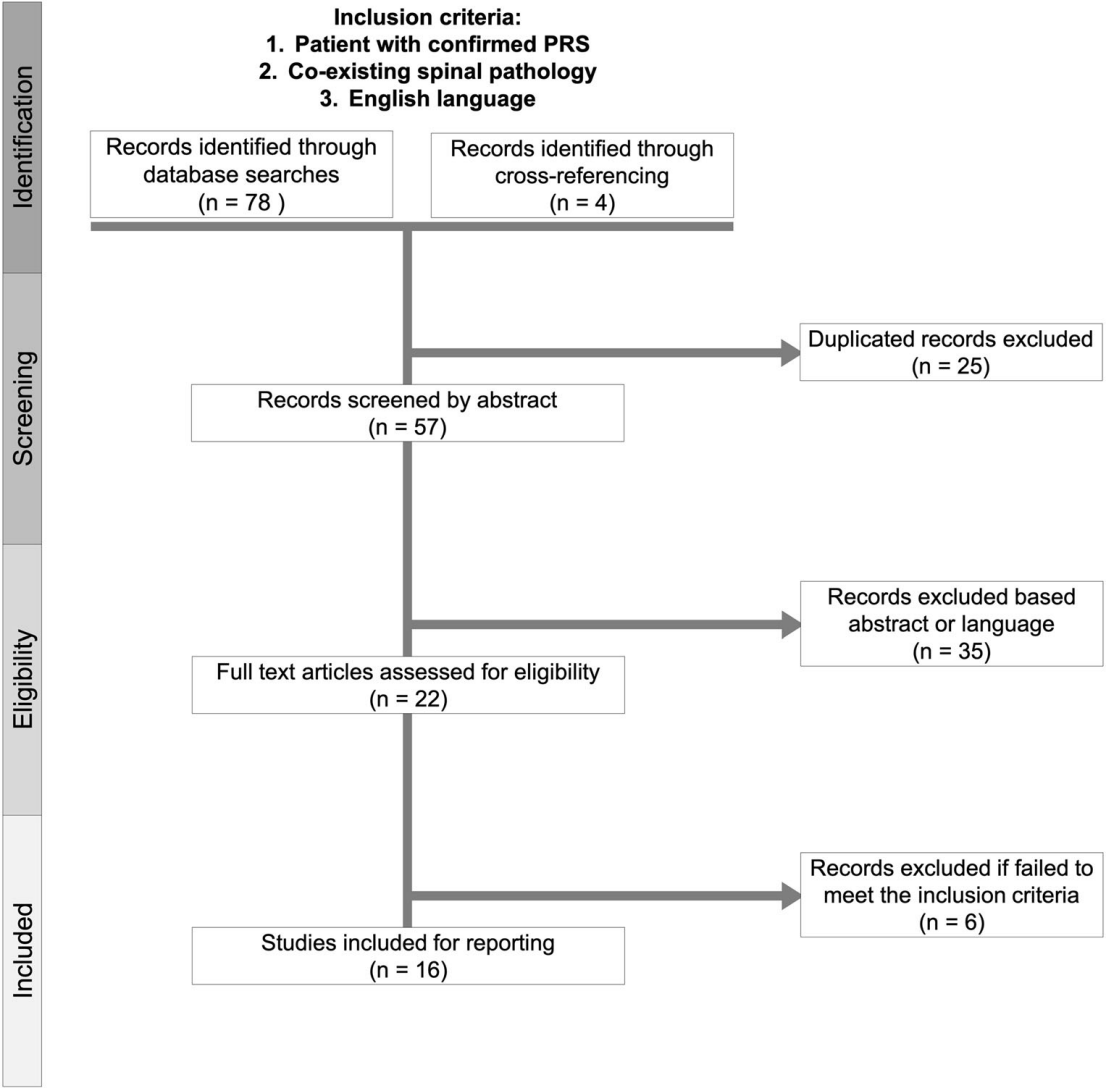
Flow diagram showing the systematic process of selecting studies included in the analysis of spinal pathologies associated with Pierre Robin sequence

We performed a systematic search for all cases of spinal diseases in patients with PRS reported in the literature using the PubMed, Google Scholar, Trip, and MEDLINE databases. The search strategy and results are summarized in Fig. 2. Inclusion criteria were (1) confirmed PRS, (2) any spinal pathology, and (3) English language. Search strings included all combinations of the terms “Pierre Robin sequence” or “Robin sequence” with the terms “spine,” “spinal,” “vertebral,” “tethered,” and “scoliosis.” Abstracts and full-text articles were screened to identify reports that passed the inclusion criteria. Additional publications were identified from the references listed in each study. We excluded reports that lacked case-level descriptions of spinal pathology, clinical characteristics, or management. The Preferred Reporting Items for Systematic Reviews and Meta-Analysis (PRISMA) criteria were followed. Ethical approval for the case report was obtained from the Institutional Review Board of Rady Children’s Hospital.

### Results

Our literature review of spinal pathology in patients with PRS identified 16 previously reported cases of spinal diseases (Table 1). The majority of patients presented with more than one spinal pathology. The most common disorders included congenital kyphoscoliosis (*n* = 7), cervical instability (*n* = 5), tethered cord (*n* = 2), and caudal regression syndrome (*n* = 2). Two patients had neural tube defects including spina bifida and myelomeningocele (*n* = 1 each). Although the majority of patients had multiple other congenital anomalies, only six patients had a known underlying genetic abnormality. Of the remaining ten patients, four had reportedly normal genetic analysis such as karyotype and/or microarray, while genetic testing was not reported in six.

**Table 1:** Results of systematic literature search for all reported cases of co-existing Pierre Robin sequence (PRS) and spinal pathologies

Case	Age	Sex	Co-existing syndrome	Presenting symptoms	Spinal pathology/management	Follow-up duration	Surgical outcome	Molecular genetics	Other anomalies/management	Reference
1	Neonate	F	Type 1 Stickler syndrome	Multiple anomalies at birth	1. Cervical instability due to congenital lack of anterior ring of C1 and lack of C2 dens/NR	NR	NA	COL.2A1 heterozygous exon 32 c.2078G>A transition	1. Micrognathia, glossoptosis, cleft palate/mandibular osteotomies 2. Stickler syndrome features 3. Sleep apnea/CPAP	[7]
2	Neonate	M	None	Multiple anomalies at birth	1. Chiari type I malformation/none 2. Caudal regression syndrome/none 3. Tethered cord/surgical release	NR	NR	Normal karyotype	1. Grade III gastroesophageal reflux/NR 2. Ventricular septal defect/NR 3. Bronchopulmonary dysplasia/NR 4. Bilateral inguinal hernia/NR 5. Multicystic dysplastic kidney/NR 6. Bilateral cryptorchism/NR 7.Bilateral short legs with malpositioned feet/NR 8. Grade I germinal matrix hemorrhage/NR	[8]
3	Neonate	F	None	Myoclonic activity and decerebrate rigidity	1. Cervical stenosis/none	NR	NA	Normal microarray	1. Micrognathia, glossoptosis, cleft palate/mandibular distraction osteogenesis	[9]
4	Neonate	M	SEDC	Multiple anomalies at birth	1. Occipitocervical instability/ C1 posterior decompression, occiput-C2 fusion, odontoidectomy	NR	NR	Normal karyotype and microarray	1. Micrognathia, glossoptosis, cleft palate/none 2. Gastroesophageal reflux/Nissen fundoplication and gastrostomy	[10]
5	2 months	M	None	Multiple anomalies at birth	1. Caudal regression syndrome/NR	NR	NA	NR	1. Micrognathia, glossoptosis, cleft palate/NR	[11]
6	3 months	M	Kniest dysplasia	Multiple anomalies at birth	1. Platispondyly and vertebral body tongue-like bony protrusions/none	1 month (mortality)	NA	NR	1. Micrognathia, glossoptosis, cleft palate/tracheostomy 2. Hydrocephalus/none	[12]
7	2 years	F	None	Quadriplegia	1. Congenital cervico-thoracic kyphosis secondary to vertebral hypoplasia/extension and closed reduction 2. Complete spinal cord injury at kyphotic level C7/T1/none	NR	NR	Normal karyotype of proband and parents	1. Micrognathia, glossoptosis, cleft palate/palatal pushback, bilateral myringotomy and tube placement 2. Speech and language delays secondary to otitis media/NR	[13]
8	8 years	M	None	Abnormal cervical range of motion	1. Occipitocervical instability of cervical spine due to ossification failure of anterior and posterior C1 arches/fusion from occiput to C2	1 yr	Complete symptom resolution	Normal karyotype	1. Micrognathia, glossoptosis, cleft palate/oral airway 2. Ventricular septal defect, heart failure/surgery, digoxin 3. Hydrocele/surgery 4. Inguinal hernia/herniorrhaphy 3. Perthes disease/varus osteotomy	[14]
9	8 years	F	None	NR	1. Congenital thoracolumbar kyphoscoliosis/external orthosis, physiotherapy 2. Atlanto-occipital and atlanto-axial subluxation/none 3. Klippel-Feil syndrome/none	NR	NA	NR	1. Micrognathia, glossoptosis, cleft palate/cleft palate repair	[15]
10	14 years	M	None	NR	1. Myelomeningocele/surgery 2. Cervico-thoracic kyphoscoliosis/NR 3. Tethered spinal cord/surgical release	NR	Complete symptom resolution	NR	1. Micrognathia, glossoptosis, cleft palate/NR	[16]
11	15 years	M	ISD	NR	1. Spinal bifida, congenital vertebral dislocation, and severe congenital scoliosis/posterior spinal fusion C6 to L1 followed by C7 to T10 anterior spinal fusion with vertebral strut graft	12 years	Iatrogenic incomplete SCI requiring decompression; worsened scoliosis	NR	1. Micrognathia, glossoptosis, cleft palate/NR 2. Iatrogenic SCI/wheelchair 3. Autism spectrum disorder, intellectual disability/NR 4. Neurogenic bowel and bladder/clean intermittent catherization 5. Sleep apnea/BiPAP and CPAP 4. Pelvic obliquity and iliac crest deformity/spinal-pelvic fusion 5. Bilateral hip subluxation and proximal femur deformity/surgery	[17]
12	15 years	M	NF2	Headache, ataxia	1. Multiple extramedullary intradural lesions, separate C7-T1 neurofibroma/NR 2. Holocord syrinx most likely due to Chiari malformation/NR	NR	NA	Deletion of 22q12.1 to 22.q12.3, including MN1 and NF2 genes	1. Micrognathia, glossoptosis, cleft palate/NR 2. Intellectual disability/NR 3. Severe bilateral hearing loss/hearing aids 4. Cerebellopontine schwannoma/resection	[18]
13	15 years	F	None	Bilateral, progressive lower limb numbness, difficulty in walking, urinary incontinence	1. Thoracic kyphoscoliosis/posterior fusion T1–T9	1 year	Complete symptom resolution	NR	1. Micrognathia, glossoptosis, cleft palate/NR	[19]
14	15 years	M	None	Occipital headache and gait disturbance	1. Chiari type I malformation/suboccipital decompression 2. Congenital occiput-C1 and C2-C3 fusions/C1 and C2 laminectomy	3 months	Complete symptom resolution	NR	1. Micrognathia, glossoptosis, cleft palate/none	[20]
15	22 years	F	12q deletion syndrome	Skeletal abnormalities, endocrine derangements	1. Thoracic scoliosis with congenital cervical and lumbar fusions/none 2. Klippel-Feil syndrome/none	NR	NA	Chromosome 12q13.2 to 12q13.3 deletion, including RPS26 and flanking genes	1. Micrognathia, glossoptosis, cleft palate/none 2. Congenital perimebranous ventricular defect/none 3. Neurodevelopmental delay/none 4. Diamond-Blackfan anemia/NR 5. Trigeminal nerve palsy/NR 6. Bilateral mixed hearing loss/NR	[21]
16	31 years	F	None	NR	1. Scoliosis/spinal fusion	NR	NR	NR	NR	[22]
17	6 months	F	10q deletion syndrome	Multiple anomalies at birth	1. Syringomyelia/none 2. Tethered cord/surgical release	1 year	Complete resolution	Chromosome 10q26.11 to 10q26.3 deletion	1. Micrognathia, glossoptosis, cleft palate/CPAP, mandibular distraction osteogenesis 2. GERD/Nissen fundoplication 3. Exotropia/medial rectus muscle recession	Present case

Age denotes the patient’s age at time of first spinal pathology diagnosis. BiPAP, bilevel positive airway pressure; CPAP, continuous positive airway pressure; F, female; FISH, fluorescence in situ hybridization; KF, Klippel-Feil; ISD, ischiospinal dysostosis; M, male; NA, not applicable; NF2, neurofibromatosis type 2; NR, not reported; SCI, spinal cord injury; SEDC, spondyloepiphyseal dysplasia congenita

Patients predominantly presented at birth or adolescence, with two patients presenting with spinal deformities at 8 years of age. Clinical presentations corresponded to the spinal levels and pathologies involved. Surgical management was pursued in nine cases (53%). The remainder of cases were managed medically. The majority of patients managed surgically had complete resolution of symptoms without neurologic complications. One patient with congenital vertebral dislocation and severe congenital scoliosis who underwent posterior spinal fusion sustained an iatrogenic spinal cord injury that caused worsened scoliosis [17]. Four patients encountered anesthesia-related challenges, including prolonged intubation postoperatively (*n* = 3) [7, 10, 16], nasopharyngeal intubation requirement (*n* = 3) [7, 16, 20], and tracheostomy requirement (*n* = 1) [10].

Tubbs et al. [8] reported the first case of tethered cord in a neonate with PRS and normal karyotype, co-occurring with several congenital anomalies including Chiari I malformation and caudal regression syndrome [8]. Abraham et al. described a 15-year-old patient with tethered cord, co-occurring with myelomeningocele and kyphoscoliosis; genetic testing was not reported [16]. Both patients were treated surgically, but outcomes were not reported.

We also performed another literature search to identify all genetic syndromes associated with PRS that are also known to predispose to spinal pathologies. These conditions are described in Table 2. Although more than 50 genetic syndromes have been associated with PRS [23], only 15 have also been associated with spinal pathologies. In 9 of these 15 syndromes, no patients have yet been reported who are affected by both PRS and spinal disease. The underlying molecular genetic disturbances predominantly involve genes responsible for extracellular matrix synthesis and organization, ribosome biosynthesis, and Golgi complex function. These genes are most commonly located on chromosomes 5, 12, and 17, with isolated cases of mutations in genomic regions 6p, 7p, and 22q. To our knowledge, ours is the first reported case of PRS and spinal pathology involving abnormalities of chromosome 10.

**Table 2:** Clinical and molecular genetic features of syndromes known to be associated with both Pierre Robin sequence and spinal pathologies

Syndrome	Gene or chromosomal region	Gene function	Spinal pathology	Other clinical features
Campomelic dysplasia	SOX9	Transcription factor, regulates chondrocyte differentiation, skeletal development and collagen formation	Scoliosis, short and flat cervical vertebrae	Short stature, campomelia, hearing loss
Stickler syndrome type I	COL2A1	Type II collagen	Spondyloepiphyseal dysplasia	Ocular, auditory, skeletal, and orofacial abnormalities
Spondyloepiphyseal dysplasia congenita	COL2A1	Type II collagen	Short spine	Pectus carinatum, myopia
Kniest dysplasia	COL2A1	Type II collagen	Kyphosis, lumbar lordosis, atlantoaxial instability	Short trunk, short limbs, reduced joint mobility
Diastrophic dysplasia	SLC26A2	Sulfate transporter; extracellular matrix organization, endochondral bone formation	Kyphoscoliosis, hypoplasia of cervical vertebral bodies, spina bifida occulta	Short stature, short limbs, joint contractures, talipes equinovarus
Cerebrocostomandibular syndrome	Unknown	Unknown	Scoliosis	Narrow thorax, rib anomalies, conductive hearing loss, growth restriction
Cerebrocostomandibular-like syndrome	COG1	Golgi complex component, glycosylation	Costovertebral defects	Microcephaly, growth restriction, developmental delay, brain anomalies, cryptorchidism
Carey–Fineman–Ziter syndrome	Unknown	Unknown	Scoliosis	Hypotonia, moebius anomaly, growth delay, feeding difficulties
Otospondylomegaepiphyseal dysplasia	COL11A2	Type XI collagen	Vertebral body anomalies	Sensorineural hearing loss, enlarged epiphyses, short limbs, typical facial features
Congenital disorder of glycosylation type IIg/CCMS	COG1	Golgi complex component; glycosylation	Vertebral anomalies	Severe micrognathia, osteopenia, rib defects (rib gaps), mental retardation, growth retardation, microcephaly
Ischiospinal dysostosis (ISD)	BMPER	Bone morphogenetic protein inhibition; osteoblast and chondrocyte regulation	Kyphoscoliosis, vertebral anomalies	Dysplasia/aplasia of ischial rami, peculiar facial morphologies
22q11.2 deletion syndrome/velocardiofacial syndrome/DiGeorge syndrome	del 22q11.2	Transcription factors; regulation of developmental processes	Upper cervical instability from odontoid hypoplasia or os odontoideum, congenital C2 to C3 fusion, dysmorphic dens	Cleft palate, cardiac anomalies, typical facies, learning disabilities
Treacher Collins syndrome	TCOF1	Ribosome biosynthesis	Dysmorphic atlas	Antimongoloid slant of the eyes, eyelid coloboma, micrognathia, microtia and other ear deformities, hypoplastic zygomatic arches, macrostomia, conductive hearing loss, cleft palate
Neurofibromatosis 2	NF2	Merlin protein involved in myelination	Extramedullary spinal tumors	Vestibular schwannomas, benign tumors of nervous system
Chromosome 12q deletion	del 12q	Transcription factors; regulation of developmental processes	Scoliosis	Developmental delay, intellectual disability, behavioral problems, and distinctive facial features

NA, not applicable

## Discussion and conclusions

Pierre Robin sequence (PRS), also known as Robin sequence (RS), begins with micrognathic mandible, leading to retro-positioning of the tongue (glossoptosis) and increased likelihood of airway compression at the level of the glottis. The inability of the base of the tongue to descend from the nasopharyngeal roof in turn impairs palate formation, leading to cleft palate [5, 24]. The triad of micrognathia, glossoptosis, and cleft palate is termed syndromic PRS (or “RS-plus”) when it occurs in association with an underlying genetic or acquired syndrome, in contrast to non-syndromic PRS occurring in isolation. Syndromic PRS carries a higher mortality than isolated PRS, making it imperative to establish definitive early diagnosis [25]. Disorders commonly associated with PRS include Stickler syndrome (most common) as well as 22q11.2 deletion, Treacher Collins, campomelic dysplasia, and Marshall syndromes. Chromosomal loci harboring genes associated with pre- and postnatal growth, neurodevelopment, morphogenesis, and patterning have been implicated in the development of PRS; these include regions 2q24.1–33.3, 4q32-qter, 17q21–24.3, and 11q21–23.1, among others [26–33].

The molecular genetic understanding of PRS has primarily been derived from case reports or heterogeneous cohorts ranging from 66 to 191 subjects [4, 33–35]. These studies have been critical to elucidating the epidemiology of PRS but are limited by lack of uniformity in genetic testing of patients and family members. Patients predominantly undergo karyotyping by conventional methods or microarray analysis. Although these approaches are sensitive for detecting copy number gains and losses associated with chromosomal imbalances, they are unable to detect point mutations, mosaic conditions, small deletions or duplications, balanced structural rearrangements, or certain polyploidy patterns. Only a small minority of patients in existing cohorts have received targeted or whole exome sequencing (WES). Studies describing systematic next-generation genetic sequencing of PRS patients or family members remain lacking.

Conducting such studies is inherently challenging due to the low prevalence as well as clinical and genetic heterogeneity of PRS. In this context, recent studies of rare neurodevelopmental disorders such as congenital hydrocephalus (CH) serve as roadmaps for future investigation. For example, Kahle and colleagues have performed parent-offspring trio WES in CH cohorts of similar size to published PRS cohorts. This unbiased strategy has uncovered that a significant portion of CH cases are associated with mutations in a defined subset of genes involved in brain development. These insights have in turn enabled hypothesis-driven laboratory investigation to guide targeted therapy development, as well as molecularly guided clinical classification that could guide surgical treatment decisions [36].

In the absence of a large genetically sequenced cohort of PRS patients, retrospective clinical series may serve as de facto cohorts to illuminate the molecular underpinnings of this disorder and its relation to other pathologies of interest. Accordingly, our systematic review and case description consolidate the current understanding of the embryological and developmental processes that may be shared between PRS and spinal disorders.

Given that PRS is commonly caused by disorders of morphogenesis, it is unsurprising that the majority of spinal pathologies we found were structural in nature and often due to disorders of bone and cartilage development. Several cases were due to genetic abnormalities in genes such as COL2A1 that are critical for chondrogenesis (Table 1). Expression of these genes is regulated by a common transcription factor, SOX9, which has independently been associated with both PRS and spinal deformity (Table 2). Similarly, we identified another report of a patient with ischiospinal dysostosis (ISD), a genetic disorder due to defects in separate pathways regulating chondrogenesis (Table 1). Several patients in our series manifested signs of cervical instability and craniocervical junction pathology, likely due to the shared developmental programs regulating formation of the cervical and mandibular regions.

We identified three patients in our literature review with both Chiari type I malformation and PRS (patients 2, 12, and 14 in Table 1). Intriguingly, one of these cases occurred in the setting of neurofibromatosis type 2 (NF2) (patient 12). Another patient had phenotypic features similar to DiGeorge syndrome (patient 2), while the final patient had congenital craniocervical vertebral fusion anomalies (patient 14). Given that NF2 and DiGeorge syndromes occur due to mutations at adjacent loci on the long arm of chromosome 22, we speculate that genes in this region may be responsible for shared developmental programs underlying morphogenesis of the mandible and spine, ultimately leading to PRS and disorders of the craniocervical junction, respectively. For example, preclinical studies have shown that the TBX1 gene on chromosome 22q11.2 is expressed in the pharyngeal arches, pouches, vertebral column, and tooth bud [23]. In this context, it is possible that deeper genetic characterization of the aforementioned patients may have revealed mutations in coding regions or regulatory elements of genes in these regions. Although neurofibromatosis type I (NF1) is also known to be associated with Chiari type I malformation [37], we did not encounter any patients with NF1 and PRS in our literature review (Table 1), nor any previously described association between NF1 and PRS (Table 2). However, the SOX9 and COG1 genes that underlie several developmental syndromes associated with both PRS and spinal pathologies are located in proximity to the NF1 gene on the long arm of chromosome 17. It is thus possible that, as in the case of NF2, patients with genetic abnormalities leading to NF1 and Chiari I malformation may also have increased rates of PRS.

We found two prior reports of tethered cord co-occurring with PRS (Table 1, patients 2 and 10). Genetic analysis and diagnostic information regarding underlying syndromes were not reported. Both patients had multiple spinal deformities such as scoliosis that are known to be associated with tethered cord. Although tethered cord has not previously been linked to the terminal region of chromosome 10q deleted in our patient, she displayed many phenotypic features previously associated with this segment, including facial abnormalities, growth and psychomotor delay, and digital anomalies [38]. Deletions of 10q26 have been reported in less than 30 prior cases; this segment contains multiple genes and regions critical for a variety of developmental processes [39] but has not previously been associated with PRS or tethered cord. It remains unclear which specific gene(s) in this region or deleted in our patient’s case (listed in Supplementary Table 1) may be responsible for either condition.

Roberti and colleagues described a 22-year-old patient with PRS and spinal deformity who harbored an interstitial chromosome 12q microdeletion covering more than 20 known genes and causing a complex malformative syndrome [21]. As in our case, genotype-phenotype correlations were not clear due to the dearth of reported similar cases and multiple genes affected. These included genes involved in extracellular matrix synthesis and cellular processes such as vesicular trafficking, similar to the other patients in our series. Collectively, these cases highlight the need for obtaining early genetic testing, establishing individualized genotype-phenotype correlations, and carefully evaluating patients for signs of underlying spinal pathology such as sacral dimples, clubfoot deformities, and frequent urinary tract infections. Detecting conditions such as tethered cord requires a high degree of clinical suspicion in patients with complex syndromes that may mask classic presenting symptoms early in the disease course.

Structural conditions such as occipitocervical instability, Chiari I malformation, and Klippel-Feil syndrome were highly represented in our literature review among reported PRS cases with spinal pathologies present at birth (Table 1), suggesting dysregulation of a common embryological process(es) underlying mandibular and spinal development. We speculate that this may take place as early as the sixth week of embryogenesis. During this week, neural crest-derived cells form the osteogenic membrane of the mandible [23], while concurrently the notochord and neural tube induce chondrification of developing vertebral structures [40]. In both of these phenomena, intercellular signaling leads to synthesis of collagen and proteoglycans, the two primary components of cartilage, which in turn serves as the “mold” for bone formation [41]. As stated above, the SOX9 transcription factor regulates many steps in chondrification including the expression of several genes involved in collagen and proteoglycan synthesis [23]. Collectively, these observations suggest that SOX9 pathway mutations leading to disordered chondrification during the sixth developmental week may be a parsimonious genetic explanation for the concomitant development of PRS and congenital structural spinal pathologies.

The management of PRS requires multidisciplinary collaboration, particularly in the setting of underlying genetic syndromes. Our systematic review and case description demonstrate that spinal pathologies are important but understudied contributors to disease burden in this patient population. Our findings raise the possibility that in patients with both PRS and spinal disorders, the range of predisposing syndromes and mutations may be more defined than previously anticipated, with pathways such as SOX9 of particular importance. Clinical screening may be most critical during specified ages such as the neonatal and adolescent periods, with emphasis on longitudinal assessment of deformity, cervical stability, and tethered cord symptomatology. Providers must encourage early genetic testing and counseling, including both microarray karyotyping and next-generation sequencing; the latter may involve whole exome sequencing for research purposes, in order to uncover novel regions of interest such as the terminal 10q deletion in our patient. Ultimately, these efforts will lead to development of targeted panels for deep sequencing at the individual level. However, developing clinically meaningful screening and management recommendations will require cohort-level genetic testing of patients and family members with rigorous genotype-phenotype correlation. In this context, the limitations of existing genetic testing, both in terms of sensitivity and clinical utility, must also be clearly discussed with patients and families.

## Electronic supplementary material

ESM 1 (DOCX 15 kb).

## Data Availability

All data collected, generated, and analyzed in this study are included in the published article and accompanying Supplementary information.

## Abbreviations

OMIM: Online Mendelian inheritance in man
PRS: Pierre Robin sequence
WES: Whole exome sequencing
