# Supplementary material for: Clinical and genetic characterization of patients with Pierre Robin sequence and spinal disease: review of the literature and novel terminal 10q deletion
Source: Childs Nerv Syst. 2020 May 12;36(7):1367–77. doi: 10.1007/s00381-020-04642-2 (PMC7300078; doi:10.1007/s00381-020-04642-2)
Supplement: Supplementary file 1 — (DOCX 15 kb). [file 381_2020_4642_MOESM1_ESM.docx]

**Supplementary Table 1.** Neonatal microarray analysis disclosed a terminal chromosome 10q deletion spanning 14.34 Mb, containing 62 Online Mendelian Inheritance in Man (OMIM) genes.

| GRK5  TIAL1  BAG3  INPP5F  MCMBP  WDR11  FGFR2  NUBP2  NSNCE4A  TACC2  PLEKHA1  ARMS2  HTRA1  DMBT1  CUZD1  PSTK  IKZF5  ACADSB  HMX3  HMX2  BUB3  GPR26  CHST15  OAT  LHPP  FAM53B  ABRAXAS2  ZRANB1  CTBP2  NMP21  UROS  BCCIP  DHX32  FANK1  ADAM12  DOCK1  FAM196A  NPS  FOXI2  PTPRE  MKI67  MGMT  EBF3  GLRX3  PPP2R2D  BNIP3  JAKMIP3  DPYSL4  INPP5A  NKX6-2  ADGRA1  KNDC1  UTF1  VENTX  ADAM8  CALY  PRAP1  ECHS1  PAOX  SPRN  CYP2E1  SYCE1 |
| --- |
